# Disparities in Comprehension of the Obstetric Consent According to Language Preference Among Hispanic/Latinx Pregnant Patients

**DOI:** 10.7759/cureus.27100

**Published:** 2022-07-21

**Authors:** Rose L Molina, Emily Adams, Ricardo Aguayo, Samantha Truong, Michele R Hacker

**Affiliations:** 1 Obstetrics and Gynecology, Beth Israel Deaconess Medical Center, Harvard Medical School, Boston, USA; 2 Obstetrics and Gynecology, Harvard Medical School, Boston, USA; 3 Obstetrics and Gynecology, Brigham and Women's Hospital and Massachusetts General Hospital, Harvard Medical School, Boston, USA

**Keywords:** pregnancy counseling, spanish, limited english proficiency, communication, informed consent

## Abstract

Background: We assessed understanding of the obstetric consent form between patients with English and Spanish language preference.

Methods: This observational study included pregnant patients who identified as Hispanic/Latinx with English or Spanish language preference (defined as what language the patient prefers to receive healthcare information) and prenatal care providers at a large academic medical center from 2018 to 2021. Patient demographics, language preference, literacy, numeracy, acculturation, comprehension of the obstetric consent, and provider explanations were collected.

Results: We report descriptive statistics and thematic analysis with an inductive approach from 30 patients with English preference, 10 with Spanish preference, and 23 providers. The English group demonstrated 72% median correct responses about the consent form; the Spanish group demonstrated 61% median correct responses. Regardless of language, the participants demonstrated limited understanding of certain topics, such as risks of cesarean birth.

Discussion: Overall comprehension of key information in an obstetric consent form was low, with differences in language groups, which highlights opportunities for improvements in communication across language barriers. Innovations in the communication of critical pregnancy information for patients with limited English proficiency need to be developed and tested.

## Introduction

In the United States, racial/ethnic disparities in obstetric care persist when controlling for social and medical factors, signaling the role of racism in driving disparities in maternal health [1-3]. Less is known regarding disparities for those whose racial or ethnic identity is compounded by limited English proficiency (LEP). According to a study of patients with LEP, those speaking non-English languages suffered approximately twice the risk of obstetric trauma compared to English-speaking patients, even controlling for race and ethnicity [4]. Variation in the risk of primary cesarean birth according to language preference has been shown among the nulliparous, term, singleton, and vertex population; we found that the risk of cesarean birth was fourfold higher for patients with Spanish preference than those with Japanese preference [5].

The availability of interpreters and language-concordant providers is often insufficient, and studies outside of obstetrics have demonstrated that patients with LEP are less likely to have fully documented informed consent and more likely to experience worse care outcomes overall [6-10]. No prior studies have investigated differences in understanding of the obstetric consent form between patients with English and Spanish preference. This is a critical area of inquiry because of the multiple procedures during delivery hospitalization and research showing that patients with LEP have limited understanding of procedures in other fields even when provided information in their preferred language [11,12]. As part of a quality assessment program, we aimed to identify disparities in communication and understanding of the global obstetric consent for childbirth care (includes all procedures and care processes during childbirth hospitalization) among patients with English and Spanish language preference. We hypothesized that patients with Spanish language preference experience more communication challenges and less understanding of the global obstetric consent than patients with English language preference.

## Materials and methods

This was an observational study of obstetric patients with English and Spanish preference at Beth Israel Deaconess Medical Center where approximately 5,000 deliveries occur annually. This study was approved by the Committee on Clinical Investigations (Protocol Number: 2017P-000561). Patients were identified through schedules of prenatal visits and ultrasound appointments. Patients were considered eligible for the study if they self-identified as Hispanic/Latinx and were pregnant up to 35 weeks' gestation. Patients were identified as having English or Spanish language preference based on their response to the question “What is your preferred language to receive healthcare information?” in the medical record. Our study team approached eligible patients at a prenatal visit or ultrasound appointment to obtain verbal consent for participation. The consent and study visits were performed in either English or Spanish, in accordance with the participant’s preferred language. If the patient enrolled, the first visit included questions about demographic information, obstetric history, level of education, access to health care, and numeracy. Additionally, the Short Assessment of Health Literacy-Spanish and English (SAHL) was used to evaluate medical literacy [13]. The Brief Acculturation Scale for Hispanics (BASH), a four-item language-based measure for Hispanic populations, was used to assess acculturation [14]. High acculturation was defined as greater than 3.0 cutoff point for BASH, while low acculturation was defined as less than or equal to 3.0 cutoff point. Throughout this study visit, questions were asked aloud to the participant, and responses were either audio-recorded and transcribed or textually recorded in real time. During the study visit, the participant had time to read the obstetric consent form in English or Spanish; participants were given the option of completing this at a second study visit if they had time constraints. The participant was given as much time as needed to review the consent form in their preferred language and was then interviewed with structured questions read aloud. Responses were either audio-recorded and transcribed or textually recorded in real time. The interview captured comprehension and areas in need of clarification in each section of the obstetric consent form: Labor, Pain Relief for Labor, Inducing Labor, Vaginal Birth, Cesarean Birth, Care After Birth, Newborn Care, and Infrequent or Rare Events. Participants were asked 18 comprehension questions (14 multiple choice and four true or false). Participants also were asked to rate their understanding and preparedness for the labor and delivery process on a four-point scale. Finally, participants were asked 31 open-ended questions regarding the perceived main points and areas of confusion for all sections of the obstetric consent form. The survey was piloted with members of the research team and with native Spanish speakers to ensure understanding of each item. Compensation was given based on the preferences of the participant (cash, public transportation voucher, or parking voucher). All responses were then recorded in REDCap [15,16]. Due to the exploratory nature of the study, we did not rely on a formal sample size calculation. Rather, we aimed to enroll 30 patients in each language group to reach saturation of qualitative themes.

We performed descriptive statistics on survey item responses, including frequencies of responses for each survey item in the English and Spanish preference groups. Medians and interquartile ranges were calculated for continuous demographic variables, such as age and SAHL score. Self-reported race/ethnicity was compared to race/ethnicity categories listed in the medical record. To assess comprehension, each participant’s total number of correct answers out of 18 items was recorded and the median scores were calculated for each language preference group.

Two investigators conducted a thematic analysis of responses to the 31 open-ended questions with formal coding through an inductive approach. For participant responses regarding the main point of each consent form section, themes were developed based on the content of that section and reported in order of frequency, with additional themes generated for responses not reflecting information in the consent form. For questions regarding participant confusion with each section, themes were based on participant responses and reported in order of frequency. In addition, misconceptions regarding the content of each section were verified by the principal investigator and reported in order of frequency. Any uncertainty in assigning codes and developing themes was settled during a discussion with the principal investigator and other study staff.

Because we acknowledge that informed consent is a bi-directional process led by a clinician, we also developed a survey about provider practices regarding the obstetric consent form. The survey included items about interactions with patients with LEP, explanations provided about the consent form, and processes for obtaining informed consent. Providers with obstetric privileges received a survey via a secure REDCap link in March 2018 and three reminder emails were sent from March to June 2018. Data were summarized as counts and percentages. 

## Results

Participant demographics

From 2018 to 2021, 152 patients (80 with English preference, 72 with Spanish preference) were approached for the study. Of the 38 English preference participants who enrolled, one withdrew and seven were lost to follow-up, yielding 30 (79%) who completed the study. Of the 26 Spanish preference participants who enrolled, five withdrew from the study and 11 were lost to follow-up, yielding 10 (38%) who completed the study (Table 1). Sixty percent of both groups were enrolled at 12-24 weeks’ gestation, with 40% of the English preference group and 30% of the Spanish preference group at 25-36 weeks’ gestation. Eleven participants (37%) in the English preference group and 6 (60%) in the Spanish preference group were nulliparous. Only 10 participants with English preference (33%) and four participants with Spanish preference (40%) had seen the obstetric consent form prior to the study visit. Among the 40 participants, only one English preference participant had taken a childbirth education class. 

**Table 1:** Demographic Characteristics of Study Participants

Characteristics	English Preference, N=30, n (%)	Spanish Preference, N=10, n (%)
Age, median (interquartile range)	30 (27-35)	33 (31-34)
Gestational age at enrollment		
<12 weeks	0 (0)	1 (10)
12-24 weeks	18 (60)	6 (60)
25-<36 weeks	12 (40)	3 (30)
Nulliparous	11 (37)	6 (60)
Born outside continental US	11 (37)	9 (90)
If born outside US, years in the US		
0-5 years	2 (18)	9 (90)
6-15 years	0 (0)	0 (0)
>15 years	7 (64)	0 (0)
Declined to answer	2 (18)	1 (10)
Usually read/speak		
Spanish only	0 (0)	5 (50)
Spanish more than English	1 (3)	5 (50)
Spanish and English equally	12 (40)	0 (0)
English more than Spanish	7 (23)	0 (0)
English only	1 (3)	0 (0)
Declined to answer	9 (23)	0 (0)
Highest educational level completed		
Some high school or diploma	13 (43)	2 (20)
Bachelor’s/associate’s degree	10 (33)	5 (50)
Graduate degree	7 (23)	2 (20)
Declined to answer	0 (0)	1 (10)

In the English preference group, most participants (63%) were born in the continental US. For the 11 participants born outside the US, most participants (64%) reported more than 15 years of residency. In contrast, 90% of participants in the Spanish preference group were born in other countries and reported living in the continental US for five years or less. In the English preference group, 20 participants (68%) demonstrated high acculturation, one participant demonstrated low acculturation, and the remaining nine participants (30%) declined to answer. In the Spanish preference group, all participants demonstrated low acculturation. Participants in both groups reported their language preference as mostly consistent with the preferred language listed in their medical records.

Finally, most participants in both groups self-reported high levels of confidence with literacy in their preferred language, with a median SAHL score ≥90% for both groups. All participants in the English preference group and 90% in the Spanish preference group identified themselves as primarily responsible for making their health-related decisions. However, in the Spanish preference group, five participants (50%) also identified other primary decision makers (e.g. spouse or other family members).

Comprehension of the obstetric consent form

All participants completed 18 comprehension questions regarding the obstetric consent form. Overall, the English preference group demonstrated a median of 72% correct responses while the Spanish preference group demonstrated a median of 61% correct responses (Table 2). Regardless of language preference, participants demonstrated limited understanding of certain topics in the obstetric consent form. Only eight participants (30%) with English preference and two (20%) with Spanish preference were able to correctly identify injury to the bladder or bowels as a risk of cesarean birth. Participants also showed limited comprehension regarding reasons for antibiotics during labor and the risk of preterm labor.

**Table 2:** Comprehension of the Obstetric Consent Form

Correct Responses About Topics in the Obstetric Consent	English Preference N=30, n (%)	Spanish Preference N=10, n (%)
Median % correct	72 (61-83)	61 (53-65)
Knowledge about childbirth care		
Definition of full-term pregnancy	21 (70)	5 (50)
Incidence of pre-term labor	10 (33)	5 (50)
Care for full-term newborn	25 (83)	7 (70)
Infrequent of rare events	25 (83)	9 (90)
Right to refuse treatment	23 (77)	5 (50)
Other risks and complications	23 (77)	7 (70)
Knowledge about interventions		
Reasons for induction	17 (57)	4 (40)
Purpose of fetal monitoring	25 (83)	6 (60)
Pain relief options	25 (83)	4 (40)
Reasons for antibiotics in labor	15 (50)	5 (50)
Reasons for cesarean	25 (83)	8 (80)
Risks of cesarean	8 (27)	2 (20)
Consent for cesarean	19 (63)	10 (100)

The understanding of specific topics in the obstetric consent form also varied between groups. Participants with Spanish preference demonstrated more than a 20% lower understanding compared with participants with English preference in six questions regarding the purpose of fetal monitoring, the definition of amnioinfusion, options for pain relief in labor, the definition of labor induction, the definition of a full-term pregnancy, and, importantly, the right to refuse specific treatments. Five participants (50%) in the Spanish preference group understood that they had the right to refuse certain treatments during labor in comparison to 23 participants (77%) in the English preference group. In contrast, only 63% of English preference participants understood that by signing the consent form they would be consenting to a cesarean.

Participant comfort with signing the consent form

Most participants endorsed being very likely to read the consent document before signing it, including 77% of the English preference group and 90% of the Spanish preference group. After reading the consent form, 87% in the English preference group and 100% in the Spanish preference group reported understanding the procedures that may happen during labor and delivery very well. Slightly over half of the English preference group felt very comfortable giving permission to everything outlined in the informed consent document, with 11 (37%) feeling somewhat comfortable, and three (10%) feeling somewhat not comfortable. In the Spanish preference group, four participants (40%) felt very comfortable giving permission, four (40%) felt somewhat comfortable, and one (10%) felt both somewhat not comfortable and not comfortable at all. Less than half of the English preference group (47%) felt very comfortable consenting to a cesarean birth if it were recommended, while eight (27%) felt somewhat comfortable, four (13%) felt somewhat not comfortable, and four (13%) did not feel comfortable at all. In the Spanish preference group, five participants (50%) reported feeling very comfortable consenting to cesarean birth, four (40%) felt somewhat comfortable, and one felt somewhat not comfortable (10%). 

Open responses

Overall, participants in both groups accurately identified the main themes for all eight sections of the informed consent document, and the majority from each group did not report points of confusion regarding any of the sections. Eleven English preference participants (37%) reported points of confusion in at least one section, including the types of people and providers, such as industry representatives, that may participate in care (20%), labor induction (13%), pain management options (7%), episiotomy, care after a vaginal repair, and the sequence of events after birth (each reported by one participant, 3%). In contrast, all but one participant in the Spanish preference group denied points of confusion in the consent form; this participant expressed confusion about the role of industry representatives in care and the definition of a hysterectomy. Occasionally, patients shared misconceptions regarding the consent form. Three misconceptions reported by more than one participant included the following: future vaginal birth is impossible after a cesarean birth (reported by five English preference participants (17%)); there are no benefits to a cesarean birth (reported by one English (3%) and five Spanish preference participants (50%)); and maternal or fetal death occurs if induction of labor is not successful (reported by two English (7%) and two Spanish preference participants (20%)). 

Process for obtaining informed consent from patients with LEP

Of the 76 eligible providers, 23 (30%) completed the survey. The majority (74%) reported reviewing the obstetric consent form between 24 and 32 weeks' gestation. Nine providers reported that a physician reviewed the form with patients (39%), eight providers (35%) typically gave the patient the form to take home and sign, while the remaining six providers declined to answer. Few providers (30%) correctly identified the obstetric consent form as having five pages of information. 

Though most obstetric providers (64%) reported using formal interpreters usually or always, six (26%) reported rarely or never using formal interpreter services. Most providers (87%) endorsed rarely or never using ad hoc interpreters. Providers reported explaining similar portions of the obstetric consent form to English-speaking and LEP patients, though few reported verbally explaining over 75% of the form or having enough time to discuss all included procedures. More providers reported "never having sufficient time" with LEP patients than with English-speaking patients. Overall, few providers felt that patients understood the consent form well, though they perceived better understanding in English-speaking compared to LEP patients. Providers also noted that the use of consent forms at other hospitals differed in the inclusion of cesarean birth and the time point at which the consent was signed (e.g. on admission for delivery instead of during prenatal care). 

## Discussion

Despite growing recognition of maternal health inequities due to racism [3], this study calls attention to the critical lens of language preference on birthing experiences and outcomes, through differences in patient understanding of common procedures during labor and delivery. Our study demonstrates that in a relatively educated group of pregnant Hispanic/Latinx patients, overall comprehension of the key information presented in a global obstetric consent form was low, with key differences in those with Spanish compared to English language preference. Patients with LEP may be less able to report confusion about the form, which hinders further discussion with their provider about misconceptions. Furthermore, variation in provider counseling practices regarding the obstetric consent can further exacerbate inequities in communication with patients with LEP. This quality assessment suggests that the obstetric informed consent process may not be optimally designed for full comprehension among patients, highlighting a need for structured communication regarding the spectrum of interventions during delivery hospitalization, with sensitivity to barriers for patients with LEP. Additionally, the findings signal the importance of creating informed consent processes that account for factors that impact understanding, including education, literacy, numeracy, and language preference. Such models may include the active incorporation of patient advocates (e.g. doulas, patient navigators, and cultural brokers) who have been shown to improve experiences and outcomes for linguistic minorities [17-20], in addition to qualified interpreters and improved access to childbirth education [21].

This study interrogates the underlying purpose of the informed consent process. While the global obstetric consent serves as a checkpoint for providers to discuss expectations and common procedures during labor and delivery, challenges in the informed consent process point to the larger need for improving overall communication with patients regarding delivery hospitalization, particularly for racial/ethnic minorities, patients with low health literacy, and those with LEP [7,22-24]. Nearly every pregnant patient will receive childbirth care in a hospital where a variety of procedures may occur, often under circumstances of varying uncertainty and urgency. Therefore, it is important for providers to discuss these possible interventions in advance to address questions and mitigate harm to patients, including negative experiences during childbirth [25,26]. Previous pregnancy experiences and childbirth education classes may shape understanding of possible interventions during labor. While there were 60% nulliparas in the Spanish group compared to 37% nulliparas in the English group, only one participant from the English group had taken a childbirth education class at the time of the study. This highlights the importance of reviewing the information in the consent document, especially for nulliparas who have not engaged in childbirth education classes.

This study builds on prior findings showing that providing materials in a patient’s preferred language alone does not effectively diminish disparities in comprehension of clinical care or procedures to be performed [11,12]. Several solutions have been studied to address this persistent challenge. In patients undergoing sterilization, consent forms designed for low-literacy audiences have been shown to improve understanding of the procedure [27]. A modified interactive consent process was proven to increase overall comprehension of informed consent among low-literacy and minoritized populations. The teach-to-goal model involved supplementing reading the consent form to patients with seven comprehension questions and targeted education in an interactive process until comprehension was achieved [12].

Limitations of our study include a small sample size and difficulty enrolling patients with Spanish preference with a completion rate of 79% among those with English preference compared to 39% with Spanish preference. A Spanish-speaking research assistant joined the study in 2019 to boost outreach to patients with Spanish preference. We allowed flexibility in splitting the study into two visits to accommodate participants’ schedule preferences. We also offered a variety of compensation options to meet people’s preferences. Due to the COVID-19 pandemic, we moved to telephonic study visits. Despite multiple adjustments to enhance the recruitment of patients with Spanish preference, we encountered difficulties in recruiting and retaining those participants, with important implications for future research designed to reach populations with LEP. Our results may demonstrate response bias and social desirability bias regarding comprehension of the informed consent document, which would likely lead to overestimating comprehension rather than underestimating comprehension. The findings may not be broadly generalizable given the single-institution nature of our study. However, the principles of informed consent, readability of medical-legal forms, and the complex counseling regarding potential interventions during labor and delivery are common issues across obstetrics practices. 

The obstetric consent functions as a crucial moment in pregnancy care to ensure patient understanding of their anticipated labor and delivery experience. As a communication tool, it is fraught with limitations if not administered consistently through a health equity lens. This study highlights the importance of addressing how language preference and LEP exacerbate health inequities in populations that face worse health experiences and outcomes in obstetric care [4,28-30]. We will begin a quality improvement project to address the gaps in understanding of the obstetric consent form, including modifications that optimize how the consent form is designed and discussed with patients with LEP.

## Conclusions

Overall comprehension of key information in an obstetric consent form was low with differences in language groups despite the majority of both groups having at least a bachelor’s degree. Our findings highlight opportunities for improvements in communication regarding the spectrum of interventions during delivery hospitalization, with sensitivity to barriers for patients with LEP. 
